# Perioperative exercise and post-operative mortality in patients undergoing oncologic surgery: a systematic review and meta-analysis

**DOI:** 10.1007/s00520-025-09611-6

**Published:** 2025-06-09

**Authors:** Christine Ibilibor, Nathan R. Weeldreyer, Siddhartha S. Angadi

**Affiliations:** 1 Division of Urologic Oncology, Department of Urology, University of Virginia, Charlottesville, VA USA; 2 Department of Kinesiology, University of Virginia, Charlottesville, VA USA

**Keywords:** Perioperative exercise, Survival, Oncology, Surgery, Mortality, Systematic review

## Abstract

**Purpose:**

To evaluate the potential impact of perioperative exercise on survival and post-operative mortality in oncologic patients undergoing surgical resection.

**Methods:**

A systematic review of randomized controlled trials administering perioperative exercise in oncologic patients undergoing surgical resection published between 2000 and 2024 was performed. Embase, PubMed, Web of Science, and the Cochrane Library were searched, and outcomes of interest included recurrence-free survival, disease-free survival, cancer-specific survival, overall survival, or post-operative mortality.

**Results:**

Of the 806 articles identified, 5 met inclusion criteria, representing 650 oncologic patients receiving pulmonary (*n* = 2), hepatobiliary (*n* = 1), gastroesophageal (*n* = 1), and colorectal surgeries (*n* = 1). Of the 650 patients, 330 were randomized to a perioperative exercise intervention. Perioperative exercise was generally administered pre-operatively (*n* = 4), ranged in duration from 2 weeks (*n* = 1) to 3 months (*n* = 1) and ranged from low (*n* = 1) to high (*n* = 1) intensity. The types of exercise regimens included walking (*n* = 5), cycling (*n* = 4), or jogging (*n* = 2) either alone or in combination with nutritional and psychosocial support. In all studies, perioperative exercise improved indices of cardiorespiratory fitness. However, perioperative exercise improved disease-free survival in only one study, and post-operative mortality did not differ between perioperative exercise and control groups.

**Conclusion:**

In the reviewed studies, the use of perioperative exercise enhanced physical fitness, but did not show a significant effect on post-operative mortality. However, survival was the primary endpoint for only one of the included studies and the type of exercise regimen varied widely. Thus, our findings are limited by its sample size and highlight the lack of uniformity in perioperative exercise regimens and the need for future trials to be powered to determine the effect of exercise on long-term oncologic endpoints.

**Supplementary Information:**

The online version contains supplementary material available at 10.1007/s00520-025-09611-6.

## Introduction

Poor pre-operative physical functioning and frailty have been linked to higher risks of post-operative complications after oncologic resection [1, 2]. Thus, perioperative exercise-based interventions have gained increased attention as strategies for reducing post-operative complications by mitigating the decrease in functional status that can be associated with major oncologic surgery [3–6]. Patients who undergo radical resection for malignancies and engage in regimented moderate to high-intensity exercise in the perioperative period experience increased mobility, enhanced cardiopulmonary reserve, and physical functioning post-operatively [3, 7, 8]. Essentially, perioperative exercise allows oncologic surgical patients to meet the physiologic demands of tissue repair, improve their physical response to post-operative recovery, and return to baseline functioning [9].

As a result, perioperative exercise has been utilized in patients who undergo radical resection across cancer types such as urologic, colorectal, pulmonary, and gynecologic malignancies to enhance patient cardiovascular fitness and reduce post-operative morbidity [1, 3, 10–12]. A representative clinical trial showed that patients who underwent a 4-week exercise regimen prior to surgery for urologic malignancies experienced a 2 ml/kg/min improvement in markers of cardiopulmonary fitness compared to patients in the control group [3]. An increase in markers of cardiopulmonary fitness like peak oxygen uptake (VO_2peak_ ) of 2 ml/kg/min is considered a clinically relevant increase in VO_2peak_ that can accommodate the increased metabolic demand of post-operative recovery [5, 13, 14]. In addition, a randomized controlled trial by Licker et al. demonstrated that patients awaiting lung resection for non-small cell lung cancer who engaged in a pre-operative exercise intervention experienced a median increase of 15% in VO_2peak_ and a decrease in pulmonary complications compared to usual care [15]. Moreover, a study by Minella et al. showed that patients awaiting resection for colorectal cancer experienced an increase in their functional capacity after pre-operative high or moderate intensity training [16].

Despite the physical fitness-related and surgical benefits of perioperative exercise regimens in oncologic surgical populations, few studies have investigated the effect of perioperative exercise on post-operative death and cancer-specific and overall survival in these patients [11]. Oncologic endpoints such as post-operative mortality and survival remain key metrics by which the efficacy of therapies and their adjuncts are measured in the management of malignancies. Randomized trials and oncologic therapies that lead to changes in clinical practice often must demonstrate a positive impact on overall or cancer-specific survival as primary and secondary endpoints [17]. Moreover, measuring post-operative mortality has been cited as a key long-term assessment measure for structured exercised-based pre-habilitation programs, yet is often omitted in perioperative exercise studies [18]. Understanding the impact of perioperative exercise as an adjunct to cancer care on oncologic endpoints has important clinical implications, namely, determining the ideal form and timing for perioperative exercise that allows for the optimal benefit and potentially the highest impact on oncologic outcomes. However, this aspect of exercise research in oncologic surgical patients remains understudied, and the effect of perioperative exercise on post-operative survival remains unknown [19]. Determining the effect of exercise-based interventions on long-term oncologic outcomes is of paramount importance to help guide recommendations for their use in clinical practice. Thus, this systematic review was designed to fill these gaps by summarizing the effect of perioperative exercise employed as an intervention on post-operative mortality across disease sites for oncologic patients undergoing resection.

## Methods

### Literature search

To estimate the potential effect of perioperative exercise on post-operative mortality and survival outcomes in patients undergoing oncologic surgery, only randomized controlled trials or post hoc analyses of randomized controlled trials were searched. In addition, this review was conducted according to the Preferred Reporting Items for Systematic Reviews and Meta-analysis (PRISMA) and Cochrane Handbook for Systematic Reviews of Interventions [20, 21]. The review protocol has been registered with the International Prospective Register for Systematic Reviews (PROSPERO, CRD42022325636) *a priori*. Embase, PubMed, Web of Science, and the Cochrane Library were searched by an experienced librarian and the citations were retrieved. The search terms utilized for each database are listed in Supplementary Table 1. The references for included studies were also searched.

### Inclusion/exclusion criteria

Randomized clinical trials or post hoc analyses of randomized clinical trials in English published from March 2000 to January 2024 were included, and the PICO (Population, Intervention, Comparison, and Outcomes) criteria were used by two reviewers (CI and NRW) to determine article eligibility [22]. The population was oncologic surgical patients; the intervention was perioperative exercise alone or in combination with other perioperative measures and compared to usual care. Outcomes were recurrence-free survival, disease-free survival, cancer-specific survival, overall survival, or post-operative mortality either reported as primary, secondary, or exploratory endpoints. Perioperative exercise was defined as a structured program employing physical exercise administered by the study investigators for patients undergoing surgery for a given malignancy either in the pre- or post-operative setting. Thus, studies that described physical exercise interventions that included aerobic exercise, strength training, endurance training, anaerobic exercise, or resistance training alone or in combination were included.

Published protocols, meta-analyses, cross-sectional studies, and single-arm trials were excluded by two reviewers (CI and NRW). Studies not written in English that did not include oncologic patients, that did not include perioperative exercise in one of the intervention arms, or that did not include a control arm were excluded. Studies that did not provide post-operative mortality or survival data post-operatively were also excluded.

### Data extraction

Articles that we retrieved from our search were screened independently by two reviewers (CI and NRW) based on title and abstract, using our inclusion and exclusion criteria. In the event of disagreement between the two primary reviewers, a consensus was reached through a discussion. For this review, the potential impact of perioperative exercise on recurrence-free survival, disease-free survival, cancer-specific survival, overall survival, or post-operative mortality was evaluated. Thus, articles that referenced mortality, or survival in the title, or abstract were included for full review. Included articles were then read in full to confirm inclusion. Data related to participant age, gender, race, cancer type, clinical and pathologic tumor stage, surgery type, duration of exercise program, frequency of exercise program, adherence to exercise program, VO_2peak_, cardiopulmonary exercise testing (CPET) data, recurrence-free survival, disease-free survival, post-operative death, overall survival, cancer-specific survival, and quality of life scores were abstracted from included studies where available.

### Risk of bias

To assess the risk of bias within each included study, we utilized the Cochrane Risk of Bias Tool for randomized controlled trials. Each included article was evaluated by two reviewers (CI and NRW) based on the domains of the randomization process, allocation concealment, deviation from the intended intervention, presence of missing data, selection of the reported outcome, and outcome measurement. Each included article was given a designation of having low, high, or some concern for bias-based, using the Cochrane Risk of Bias Tool [23].

### Statistical analysis

Descriptive statistics were applied to abstracted variables. The chi-square test was used to determine the association between categorical variables. To investigate differences in post-operative mortality between exercise intervention and control groups, odds ratios (OR) and 95% confidence intervals were generated when possible. *I*^2^ statistics was used to evaluate heterogeneity among included studies. An *I*^2^ value of 0%, 25%, 50%, and 75% represented no, low, moderate, and high heterogeneity, respectively. A random-effect model was used to pool ORs. Statistical analysis was performed in R version 4.3.1, and the meta-analysis was performed, using the ‘meta’ package (R Foundation for Statistical Computing, Vienna, Austria). A *p* value < 0.05 was considered statistically significant.

## Results

### Literature search

**Fig. 1:**
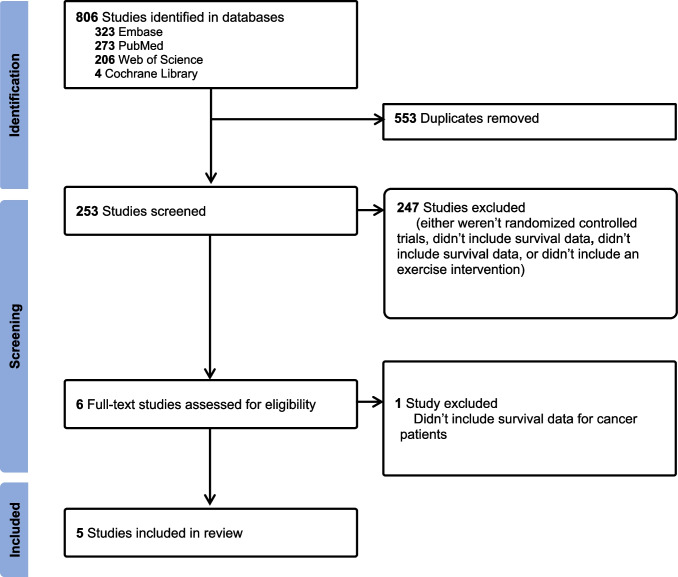
PRISMA flow diagram

Our search retrieved 806 articles. After these studies were screened by two reviewers (CI and NRW) through inspection of the titles and abstracts, 800 articles were excluded and 6 were selected for full text review [24–29]. However, one article was excluded due to the absence of survival data specific to cancer patients (Fig. 1) [25].

### Overall study characteristics

The 5 included studies represented 650 cancer patients (330 in the exercise and 320 in the usual care arms). The mean age of included participants in the exercise and usual care arms ranged from 56 to 68 and 56 to 71, respectively. Post-operative follow-up for the included studies ranged from 30 days to 5 years and represented patients undergoing pulmonary, gastroesophageal, colorectal, and hepatobiliary surgeries. One study administered exercise in the post-operative period, and employed a graduated walking program spanning 3 months [29]. The remainder of the studies employed pre-operative exercise programs that lasted 14–30 days [24, 26–28]. Three studies included perioperative exercise in combination with nutritional support and psychosocial or psychological counseling as a part of a trimodal pre-habilitation program [24, 27, 28]. However, two studies implemented exercise alone [26, 29]*.* Additional study characteristics are listed in Table 1. The overall risk of bias was low for 4 of the 5 articles (Table 2.).

**Table 1:** Included study characteristics

			Mean age (y)				Participants (***n***)				
Study	Country	Design	Ex	UC	Cancer type/surgery performed	Follow-up	Ex	UC	Timing of exercise	Description of perioperative exercise	Primary findings
Bausys et al. 2023	Lithuania	RCT	61	64	Gastric Total gastrectomy Subtotal gastrectomy Esophagectomy or palliative procedures	1 y	61	61	Pre-op	Home-based endurance, respiratory muscle, resistance training, and stretching combined with nutritional and psychological support	Exercise arm experienced 60% reduction in post-operative complications (RR: 0.40, CI 0.24 to 0.66) at 90 days compared to UC
Lui et al. 2020	China	RCT	56	56	Non-small cell lung cancer VATS lobectomy	30 days	37	36	Pre-op	Home-based aerobic exercise combined with nutritional and psychological adjustment counseling	The mean difference in the 6 MWD was 60.9 m longer in the exercise group compared to UC
Trépanier et al. 2019	Canada	Post-hoc of 2 RCT and a cohort study	68	71	Colorectal Left hemicolectomy Right hemicolectomy Subtotal hemicolectomy Transverse hemicolectomy Low anterior resection Abdominoperineal resection	5 y	104	98	Pre-op	Home-based moderate intensity aerobic exercise with resistance training combined with nutritional counseling and anxiety reduction techniques	Exercise improved 5-year disease-free survival in colorectal cancer patients with a hazard ratio of 0.45 (95% CI, 0.21–0.93)
Karenovics et al. 2017	Switzerland	RCT	64	64	Non-small cell lung cancer Bi-lobectomy Pneumonectomy Lobectomy Segmentectomy VATS	1 y	74	77	Pre-op	Facility-based HIIT cycling program	Post-operative survival at 1 year was similar at 93% vs. 91% (*p* = 0.506) in the exercise and UC groups
Yeo et al. 2012	USA	RCT	66	67	Pancreatic and periampullary Pylorus preserving pancreatico-duodenectomy Classic Whipple Distal pancreatectomy	1.58 y	54	48	Post-op	Home-based walking program	FACIT-Fatigue Scale scores were higher in the exercise group compared to UC (*p* = 0.05), and there was higher improvement in the mental health domain of the SF-36v2 in the exercise group (*p* ≤ 0.05) compared to UC

*6MWD* 6-min walk distance, *CI* confidence interval, *Ex* exercise, *FACIT* The Functional Assessment of Chronic Illness Therapy, *HIIT* high-intensity interval training, *RCT* randomized controlled trial, *RR* risk ratio, *UC* usual care, *VATS* video-assisted thoracoscopic surgery, *y* years

**Table 2:**
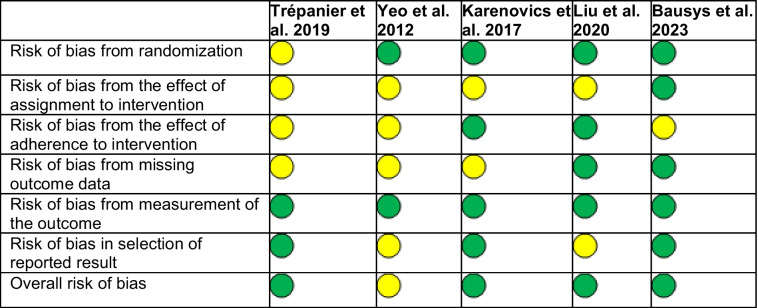
Cochrane risk of bias for included articles

Low (green), high (red) or some concern (yellow) for bias for each domain

### Exercise regimen characteristics

Each included study reported compliance with the prescribed exercise regimen at rates over 80%. Four studies utilized home-based largely unsupervised exercise [24, 27–29]. One study employed exercise performed under the supervision of a physiotherapist [26]. To titrate the intensity of the prescribed exercise regimen, four studies used heart rates in the range of 40%–90% of maximum heart rate to adjust work rates [24, 26–28]. The duration of exercise sessions in each study ranged from 30 to 38 min, and for all studies these exercise sessions were prescribed at least 3 times weekly. Exercise regimens were primarily aerobic; however, one study combined aerobic exercise with resistance training and stretching while another combined aerobic exercise with resistance training [24, 28]. The characteristics of the perioperative exercise regimens are presented in Table 3 based on the FITT (frequency, intensity, time, and type) principle.

**Table 3:** Exercise intervention characteristics

Study	Intervention period (days)	Frequency, days per week	Intensity/target HR or WR	Time, session duration (minutes)	Type	Adherence (%)
Bausys et al. 2023	30	7	Moderate, 40–65% HR_max_	60	Unsupervised home-based endurance training daily (walking, stair climbing, water exercises, or cycling) with respiratory muscle training daily with resistance training with stretching	Not reported
Lui et al. 2020	14	3	Moderate, 70% HR_max_	30	Unsupervised home-based moderate to high intensity jogging, walking, or cycling program	100%
Trépanier et al. 2019	29	3–4	Moderate, 60—70% HR_max_	30	Variably supervised home-based moderate intensity walking, cycling, or jogging program with resistance training 3–4 times weekly	80%
Karenovics et al. 2017	25	3	High 90% HR_max_, > 90%, WR_peak_	38	Supervised HIIT cycling program 3 times weekly	87%
Yeo et al. 2012	90	3–5	Low, none	10–30	Unsupervised home-based walking program	87%

*HIIT* high-intensity interval training, *HR*_*max*_ heart rate maximum, *WR*_*peak*_ peak work rate

### Impact of exercise regimen on exercise capacity

Only two studies collected VO_2peak_ pre-operatively and post-exercise [24, 26]. Of those, one study reported a 2.9 ml/kg/min increase in the mean VO_2peak_ in the perioperative exercise group compared to a 1.5 ml/kg/min decrease in the control group (95% confidence interval (CI), 1.1–4.2, *p* < 0.01) [26]. The other study demonstrated no increase in VO_2peak_ in the perioperative exercise post-exercise program [24]. Two studies reported a higher increase in the 6-Min Walking Distance (6MWD) in the perioperative exercise group following the perioperative exercise intervention compared to control [24, 27]. The Liu et al. study showed a 45.1 m vs. 3.8 m increase from baseline (*p* < 0.001) in 6MWD in the exercise and control groups, respectively. In addition, that study showed that this post-exercise intervention increase in the 6MWD was preserved 30 days post-operatively after completion of the exercise intervention (21.5-m increase vs. 36.1-m decrease). This study also reported an overall higher increase in 6MWD in the perioperative exercise group with a mean difference of 60.9 m (32.4–89.5, *p* < 0.001) between the perioperative exercise and control groups post-exercise program [27]. The Bausys et al. study showed a 31 m vs. 14 m increase from baseline (*p* = 0.001) in the 6MWD post-exercise in the exercise and control groups, respectively [24]. One study showed that participants in the post-operative exercise group were able to walk an average of 5553 ft more than the usual care group 2 months after surgery (10,772 ft vs. 5219 ft) [29].

### Exercise and quality of life

Only two studies collected quality of life metrics in the perioperative period [24, 29]. The Bausys et al. study showed a 13-point increase (95% CI, 4–21, *p* = 0.005) in the European Organization for Research and Treatment of Cancer Quality of Life Questionnaire (EORTC QLQ-C30) global health status score in the exercise group compared to no change in the control group [24]. In addition, they showed increases in the Hospital Anxiety and Depression scale in the exercise group (*p* = 0.001) compared to no change in the control [24]. Yeo et al. reported a higher improvement in the mental health domain of the Short Form-36v2 health survey quality of life questionnaire in the exercise group (*p* ≤ 0.05) compared to usual care [29].

### Exercise and post-operative mortality

**Fig. 2:**
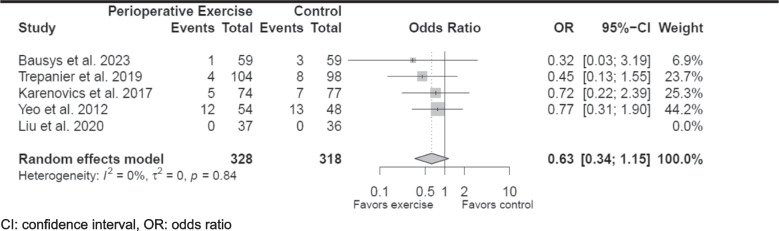
Forest plot of post-operative deaths in the perioperative exercise group compared to control groups. CI, confidence interval; OR, odds ratio

Post-operative mortality or survival were secondary or exploratory endpoints for each included study, and post-operative deaths were abstracted from each. Four studies reported the number of post-operative deaths during the follow-up period [24, 27–29]. One study reported the number of deaths at 1 year post-operatively, and one reported disease-free survival [26, 28]. Trépanier et al. determined that perioperative exercise independently improved disease-free survival in colorectal cancer patients with a hazard ratio of 0.45 (95% CI, 0.21–0.93); however, it did not affect overall survival [28]. The other included studies noted no association between perioperative exercise and post-operative mortality nor survival [24, 26, 27, 29]. One study reported no deaths during the study period [27]. The aggregate number of post-operative deaths that were reported in the perioperative exercise and control groups across included studies were similar (22 vs. 31, *p* = 0.159). While there was no heterogeneity among the included studies, there was no statistically significant association between perioperative exercise and post-operative death (odds ratio 0.63, 95% CI, 0.34–1.15) (Fig. 2). However, none of the included studies were powered to determine the association between survival and perioperative exercise.

## Discussion

This systematic review examines the association between perioperative exercise in the form of aerobic exercise, strength training, endurance training, anaerobic exercise, or resistance training alone or in combination and post-operative mortality in oncologic patients undergoing surgical resection. Our findings show that fewer post-operative deaths were reported in perioperative exercise group compared to usual care (22 vs. 31, *p* = 0.159); however, this difference was not statistically significant. Despite this, our review is a novel investigation that has coalesced all available data across cancer types on key oncologic endpoints such as post-operative mortality and disease-free survival in the post-operative period among surgical patients receiving perioperative exercise. To our knowledge, this study is the first of its kind to evaluate the relationship between post-operative oncologic outcomes and perioperative exercise regimens in randomized controlled trials across cancer types in a surgical population. The likely reason for the rarity of our study is the short time period between diagnosis and surgical intervention as well as the prolonged post-operative period for these major cancer surgeries [8, 12]. Our study also highlights the gaps in the literature as none of the included articles used post-operative survival, or death as a primary outcome.

Studies have shown that physical exercise, both strength training and aerobic, are associated reduced cancer-related mortality in non-surgical oncologic patients [19, 30, 31]. A review demonstrated that physical exercise significantly reduces the risk of mortality among oncologic patients with a relative risk of 0.76 (95% CI, 0.40–0.93, *p* = 0.009) [19]. In addition, a meta-analysis showed that any amount of resistance training such as weight lifting was associated with a 14% relative reduction in cancer mortality (relative risk 0.86, 95% CI, 0.78–0.95) among patients who were not undergoing surgery [31]. Thus, it is conceivable for increases in physical activity among surgical oncologic patients, through perioperative exercise programs, to contribute to improved post-operative survival. Reported mechanisms by which exercise can positively impact survival in oncologic patients are improved tolerance and enhancement of cancer related therapies, as well as reduction in treatment-related adverse side effects [32]. Within this context, a systematic review by Yang et al. demonstrated that one of the biologic mechanisms by which exercise can improve the efficacy of systemic therapy is through the proangiogenic nature of repeated bouts of exercise which enhances the delivery of chemotherapeutics to tumor tissue [33]. Moreover, a review by Bland et al. reported higher chemotherapy completion rates among breast cancer patients who engaged in exercise while receiving chemotherapy [34]. In our review, perioperative chemotherapy completion rates were not reported, and studies documenting the relationship between perioperative exercise chemotherapy tolerance remain limited in surgical patients with one study reporting improved chemotherapy completion rates prior to surgery in esophageal cancer patients [35]. Thus, the impact perioperative exercise on survival as it relates to chemotherapy tolerance and efficacy warrants further evaluation in future large-scale studies.

While the factors that affect post-operative survival in oncologic patients are multifactorial and often tumor stage-dependent, perioperative physical functioning can serve as an important prognostic marker for survival in surgical patients [36]. A prospective cohort study by Brown et al. reported a 21.4% risk difference in 3-year disease free survival among stage III colorectal cancer patients who reported ≥ 1.5 h/week of light to moderate intensity recreational exercise after surgical resection compared to those who did not (87.1% vs. 65.7%, 95% CI, 9.2–37.1, *p* < 0.001) [36]. Our study confirms that perioperative exercise can enhance pre- and post-operative physical functioning in surgical oncologic patients as each included study demonstrated an improvement in aerobic capacity parameters like 6MWD and VO_2peak_ after the exercise regimen. Thus, we show that perioperative exercise as an intervention can serve as a prescriptive measure to bolster physical activity. However, we were unable to determine whether these improvements in aerobic capacity translate to improvements in long-term oncologic endpoints. Thus, further studies in longitudinal studies appropriately powered to discern this are necessary.

We also demonstrate that while the field of perioperative exercise for cancer patients continues to grow across disease types, there is still opportunity for standardization of exercise interventions. We show that there is significant variability in the duration and intensity of these prescribed exercise regimens which speaks to the lack of standardization in this perioperative approach. For example, in our review, programs lasted 2 weeks to 3 months. Moreover, each study employed aerobic regimens; however, the intensity of the exercise prescription varied widely from low impact low intensity walking programs to supervised cycling-based high-intensity interval training (HIIT). This lack of standardization in exercise intensity and duration may contribute to our inability to demonstrate a direct association between post-operative survival and perioperative exercise.

The use of prescriptive aerobic exercise as an adjunct to the surgical care of oncologic patients has wide clinical and public health implications. Given the known health benefits of aerobic exercise, perioperative exercise has the potential to enhance the global health of oncologic surgical patients by utilizing surgical care as a key entry point for increasing their physical activity long-term [37, 38]. Thus, our study builds on the growing literature surrounding the physiologic benefits of perioperative exercise; however, this review also highlights how the oncologic benefits remain unclear.

Some limitations of our review include the heterogeneity in the exercise regimens of the included studies which prevented direct comparison of exercise-based outcomes between studies. Other limitations include its small sample size of only 5 studies representing 650 patients with relatively few reported post-operative deaths which could contribute to the absence of a measurable association between perioperative exercise, post-operative mortality, and survival.

## Conclusion

The use of perioperative exercise is emerging as an important adjunct to the surgical care of oncologic patients. Continued study is necessary to determine the ideal timing, duration, frequency, and intensity of prescribed regimens. Despite the clear physiologic benefits of perioperative exercise, determining its association with oncologic outcomes is necessary prior to employing it in clinical practice on a large scale as a key cancer care intervention. Future clinical trials investigating the utility of perioperative exercise will be powered to ascertain differences in cancer specific outcomes. To the degree that markers of survival guide clinical practice, clinicians and surgeons will require targeted studies on the association between post-operative survival and perioperative exercise to guide patient counseling on its use.

## Supplementary Information

Below is the link to the electronic supplementary material.

Supplementary file1 (DOCX 14 KB)

## Data Availability

The data used in this manuscript is publicly available.
