# Supplementary material for: Perioperative exercise and post-operative mortality in patients undergoing oncologic surgery: a systematic review and meta-analysis
Source: Support Care Cancer. 2025 Jun 9;33(7):558. doi: 10.1007/s00520-025-09611-6 (PMC12146225; doi:10.1007/s00520-025-09611-6)
Supplement: Supplementary file 1 — Supplementary file1 (DOCX 14 KB) [file 520_2025_9611_MOESM1_ESM.docx]

| **Supplemental Table 1.** Search Strategy | | |
| --- | --- | --- |
| **Database** |  | **Results** |
| PubMed | ((((exercise[mesh] OR exercise therapy[mesh] OR preoperative exercise[mesh] OR exercis*[tiab] OR aerobic*[tiab] OR running[tiab] OR swimming[tiab] OR "high intensity interval training"[tiab]) AND (((("Neoplasms"[Mesh]) AND (surgery[MeSH Subheading])) OR (Surgical Oncology[mesh])) OR "Neoplasms/surgery"[Mesh] OR mastectomy[tiab] OR (surgery[tiab] AND oncolog*[tiab]) OR (surgery[tiab] AND cancer[tiab]))) AND (prehabilitat*[tiab] OR periop*[tiab] OR preoperat*[tiab] OR "pre operat*"[tiab] OR "preoperative care"[mesh]))) AND (randomized controlled trial* AND (english[Filter])) | 273 |
| Embase | ('exercise'/exp OR 'kinesiotherapy'/exp OR 'preoperative exercise'/exp OR 'exercis*':ti,ab,kw OR 'aerobic*':ti,ab,kw OR 'running':ti,ab,kw OR 'swimming':ti,ab,kw OR 'high intensity interval training':ti,ab,kw) AND ('neoplasm'/exp AND 'surgery' OR 'surgical oncology'/exp OR 'neoplasm'/exp/dm_su OR 'mastectomy':ti,ab,kw OR ('surgery':ti,ab,kw AND 'oncolog*':ti,ab,kw) OR ('surgery':ti,ab,kw AND 'cancer':ti,ab,kw)) AND ('prehabilitat*':ti,ab,kw OR 'periop*':ti,ab,kw OR 'preoperat*':ti,ab,kw OR 'pre operat*':ti,ab,kw OR 'preoperative care'/exp) AND 'randomized controlled trial*' AND 'english' | 323 |
| Web of Science | ((((TS=(oncolog* or cancer or neoplasm)) AND TS=(surgery or surgical)) AND TS=(exercis* or high intensity or aerobic)) AND TS=(preoperative or perioperative or prehabilitation)) AND ALL=(randomized controlled trial* ) | 206 |
| Cochrane Reviews | (oncologic or cancer or neoplasm) AND surgery AND (exercise or high intensity or aerobic) AND (preoperative or perioperative or prehabilitation) | 4 |
